# Disorganization in individuals at clinical high risk for psychosis: psychopathology and treatment response

**DOI:** 10.1007/s00406-024-01855-3

**Published:** 2024-06-25

**Authors:** Arianna Biancalani, Michele Occhionero, Emanuela Leuci, Emanuela Quattrone, Silvia Azzali, Giuseppina Paulillo, Simona Pupo, Pietro Pellegrini, Marco Menchetti, Lorenzo Pelizza

**Affiliations:** 1 Department of Biomedical and Neuromotor Sciences, “Paolo Ottonello” Psychiatry Institute, “Alma Mater Studiorum” Università degli Studi di Bologna, Via Pepoli 5, Bologna, 40123 Italy; 2 Department of Mental Health and Pathological Addictions, Azienda USL di Parma, Largo Palli 1/a, Parma, 43100 Italy; 3 Department of Mental Health and Pathological Addictions, Azienda USL-IRCCS di Reggio Emilia, Via Amendola 2, Reggio Emilia, 43100 Italy; 4 Division of Pain Medicine, Department of Medicine and Surgery, Azienda Ospedaliero-Universitaria di Parma, Via Gramsci 14, Parma, 43100 Italy; 5 Department of Mental Health and Pathological Addictions, Azienda USL di Bologna, Via Castiglione 29, Bologna, 40124 Italy

**Keywords:** Disorganization, Clinical high risk for psychosis, Early psychosis, Early intervention, Follow-up, Antipsychotic

## Abstract

Disorganization is a nuclear dimension of psychosis, especially in schizophrenia. Despite its relevant association with poor prognosis and negative outcomes, it is still under-investigated compared to positive and negative symptoms, in particular at the onset of illness. This study explored disorganization in youth at Clinical High Risk for Psychosis (CHR-P) over a 2-year period. A sample of 180 CHR-P participants (50% males; 51.1% with baseline second-generation antipsychotic medication) recruited within a specialized CHR-P service completed the Positive and Negative Syndrome Scale (PANSS) and the Global Assessment of Functioning (GAF) scale. Across the follow-up, we examined key associations of disorganization with other domains of psychopathology, functioning, and treatment response using Spearman’s rank correlation coefficients and linear regression analyses. Our results showed a significant longitudinal reduction in disorganization severity levels across the follow-up. This decrease was significantly associated with improvements in negative symptoms and daily functioning, with a shorter duration of untreated psychiatric symptoms, and with baseline equivalent dose of antipsychotic medication. No significant longitudinal associations with other treatment component of the PARMS program were found. Our findings suggest a longitudinal improvement in disorganization dimension in CHR-P individuals, especially in the context of early interventions targeting reduction in the duration of untreated psychiatric symptoms and favoring a prompt antipsychotic therapy.

**Supplementary Information:**

The online version contains supplementary material available at 10.1007/s00406-024-01855-3.

## Introduction

*Disorganization* is a common and easily misused term in everyday clinical practice [1] As a symptom mostly associated with *schizophrenia spectrum disorders*, it is currently used for the description of a broad range of manifestations, concerning disturbances in behavior, thought and speech [2]. Dating back to Bleuler [3], the core definition of schizophrenia itself was built around the recognition of a disease where thought (associations), emotions and behavior (abnormal affect and ambivalence) lost coordination and coherence. Consequently, the term “disorganized” acquired mildly different meanings, as a descriptor.

However, the first conceptualization of the now-called “disorganization” features in psychosis can be found in the classical descriptions of “*heboidophrenia*” [4] and “*hebephrenia*” [5], two forms of psychosis classically having their first appearance during puberty. Their clinical presentation was characterized by silly behavior, inappropriate affect, hardly systematized delusions/hallucinations, and thought disorder (especially in heboidophrenia) [6, 7]. Within a decade, hebephrenia and heboidophrenia were firmly embedded concepts of adolescent insanity [8].

Some years later, Kraepelin [9] included hebephrenia as a specific subgroup of his “dementia praecox”. His effort to create a unitary model of psychosis inevitably led to the loss of what the term “hebephrenia” was meant to describe in a specific way (e.g., the close link to puberty and adolescence), yet determined the first relevant conceptualization of the *disorganization syndrome* [10]. In line with Kraepelin, hebephrenia was included among schizophrenia subtypes until the 5th edition of Diagnostic and Statistical Manual of mental disorder (DSM-5) [11], where all the subgroups were eliminated. What led to the removal of these categories was the increasing evidence that, despite their historical and theoretical meaning, they had low stability and reliability [12].

However, although considered by classical psychopathology as a “core” feature of psychosis (especially schizophrenia), disorganization more recently drew *less attention* than positive and negative symptoms [13]. This was probably because of early models of schizophrenia psychopathology incorporating disorganization with “reality distortion” features (i.e., delusions and hallucinations) to establish a positive domain [14]. To date, the conflation of positive and disorganized symptoms still persists, as can be observed in the most commonly used psychopathological instruments for assessing psychosis psychopathology (such as the Positive and Negative Syndrome Scale [PANSS]) [15, 16]. Additionally, most of the current measures of disorganization in psychosis research were derived from factor analyses based on the dichotomic (positive vs. negative) model of symptoms [17] and were extrapolated within psychometric tools not specifically developed for the assessment of disorganization. Indeed, some items of these statistical dimensions are not focused on disorganized features (e.g., the PANSS “Disorientation” item) [18].

Lastly, as evidence reported that disorganization in psychosis is a clinical predictor of poor prognosis and daily functioning deterioration [19], first-line treatments in “Early Intervention in Psychosis” (EIP) programs should also consider this crucial psychopathological target, especially at the illness onset [20]. In this respect, most research on disorganization was carried out in clinical populations with prolonged psychosis, having cross-sectional design. Empirical investigations on disorganized symptoms in *early stages* of psychosis (including at-risk mental states) are scarce, and a lack of knowledge still affects their longitudinal course and their treatment response.

Based on this background, the *aims* of this study were: (a) to investigate the longitudinal stability of disorganization in young individuals at Clinical High Risk for Psychosis (CHR-P) across a 2-year follow-up period, and (b) to examine any significant association of disorganization with sociodemographic characteristics, clinical features, and the specialized treatment components of an EIP program across the follow-up. As differences in disorganization and treatment outcome could be just a proxy of those CHR-P individuals who has been on Antipsychotic (AP) drug at the time of inclusion or how much medication someone already received, we also decided to compare CHR-P subjects with and without baseline medication in terms of sociodemographic and outcome information, and most importantly, symptomatology, especially disorganization. To the best of knowledge, no longitudinal investigation specifically examining disorganization in CHR-P individuals has been reported in the literature to date.

## Methods

### Subjects and setting

All participants were consecutively recruited within the “Parma At-Risk Mental States” (*PARMS*) program from January 2016 to December 2020. The PARMS program was a specialized “Early Intervention in Psychosis” infrastructure diffusely implemented in all adult and adolescent mental healthcare services of the Parma Department of Mental Health (Northern Italy) [21].

*Inclusion criteria* were: (1) specialized help-seeking request; (2) age 12–25 years, (3) to meet CHR-P criteria at entry, as defined by the “Comprehensive Assessment of At-Risk Mental States” (CAARMS): i.e, “Genetic Vulnerability” (GV), “Attenuated Psychotic Symptoms” (APS), or “Brief Limited Intermittent Psychotic Symptoms” (BLIPS) [22].

*Exclusion criteria* were: (1) past full-blown (non-affective or affective) psychotic episodes; (2) past exposure to AP medication or current AP prescription for more than four weeks, (3) known intellectual disability (IQ < 70); (d) neurological or other somatic disorder with psychiatric manifestations (including traumatic brain injury). Past AP use (i.e., in past illness episode before the PARMS enrollment) was considered as a proxy for a past psychotic episode, consistently with the original CAARMS criteria for psychosis threshold [23]. A current AP use for more than four weeks (i.e., in the present illness episode at the PARMS enrollment) was required to minimize pharmacological interference with baseline assessment within our EIP program [21]. However, no dosage threshold was selected.

All CHR-P participants and their parents (if minors) agreed to participate in the research and gave their written informed consent prior to their inclusion in the study. Local relevant ethical approval was obtained for the research (AVEN Ethics Committee: protocol n. 559/2020/OSS*/AUSLPR). This investigation was carried out in accordance with the Code of Ethics of the World Medical Association (1964 Declaration of Helsinki and its later amendments). The data supporting the findings of this research are available on reasonable request from the corresponding author. The data are not publicly available due to privacy and/or ethical restrictions.

### Assessment

The psychopathological assessment included the CAARMS, the PANSS, and the “Global Assessment of Functioning” (GAF) scale [11].

The *CAARMS* is a semi-structured clinical interview developed to cover various aspects of attenuated psychopathology. The CAARMS “*Positive Symptoms*” subscale was used to define both CHR-P and psychosis threshold criteria. Specifically trained PARMS team members conducted CAARMS interviews at entry, using the approved Italian version (CAARMS-ITA) [24]. Regular CAARMS supervision sessions and scoring workshops were implemented and maintained to ensure good interrater reliability values [25].

The *PANSS* is a widely used structured clinical interview for assessing psychopathology in patients with psychosis, including young individuals with early psychosis [26, 27].

As for how to *measure disorganization*, we preliminarily examined the goodness of fit of two different PANSS “Disorganization” factor solutions previously identified in the literature on individuals with psychosis or at CHR-P, using a Confirmatory Factor Analysis (CFA): (1) the 8-item “disorganization” factor model (including PANSS P2, N5, N7, G5, G10, G11, G13, and G15 items) proposed in a meta-analysis on the PANSS by Shafer and Dazzi [18], and (2) the 5-item factor model (including PANSS P2, P5, G9, G11, and G13 items) identified by Yang and co-workers [28] in a specific CHR-P population in Singapore.

The *GAF* is a widely used scale to assess clinical and socio-occupational outcomes in individuals with severe mental disorders, including young individuals with early psychosis [29] and CHR-P populations as well [30].

A sociodemographic and clinical chart (including information on ethnic group, gender, age, years of education, past specialist contact, family history of psychosis, current substance abuse, and “Duration of Untreated Psychiatric Symptoms” [DUPS, defined as the time interval [in weeks] between the onset of any psychiatric symptom in need of specialist treatment and initiation of an adequate, specialized psychopharmacological/psychosocial intervention]) [31] was completed at entry. Specifically, the DUPS was based on interviews with the individual and at least one key informant (usually a close relative), and was specifically referring to the time interval before the first psychiatric contact and before the first psychotropic prescription.

The initial DSM-5 diagnosis was formulated through assessments conducted by a minimum of two trained PARMS team members, using the Structured Clinical Interview for DSM-5 disorders (SCID-5) [32]. All psychopathological instruments were administered both at entry and every 12 months during the 2-year follow-up period. Information on AP dose and intensity of the different components of PARMS psychosocial interventions was collected both at baseline and every 12 months across the follow-up.

### Procedures

After CAARMS interviews and baseline psychopathological assessment, CHR-P participants were assigned to a *multi-disciplinary team* (consisting of a clinical psychologist, an early rehabilitation case manager, and a psychiatrist) within 3–4 weeks (see Supplementary Materials [Table S1] for details on treatment components of the PARMS program). In accordance with current official guidelines on EIP [33, 34], *AP* drug prescription should be proposed when individuals at CHR-P (a) showed a sudden decline in daily functioning, (b) had a rapid escalation to overt psychotic symptoms, (c) showed an immediate risk of suicide or severe violence, or (d) did not adequately respond to any other psychosocial interventions. As a first-line pharmacological treatment, low-dose second-generation AP medication was indicated [35]. Antidepressant and benzodiazepine medication could be prescribed for anxiety, depressive symptoms, and/or insomnia.

After having examined the goodness of fit for both PANSS “Disorganization” factor solutions in the CHR-P total sample using CFA, we investigated any relevant associations of disorganized dimension with sociodemographic, clinical and other psychopathological parameters both at baseline (T0) and across the follow-up. Finally, we examined any significant longitudinal changes in disorganization severity levels over time and their potential associations with treatment components of the PARMS program. As baseline and longitudinal changes in disorganization severity levels could be simply confounded by the fact of how far along CHR-P subjects was in the treatment trajectory, we also conducted our statistical analyses in baseline AP medicated and unmedicated CHR-P subgroups separately.

### Statistical analysis

Data were analyzed using the Statistical Package for Social Science (SPSS) for Windows, version 15.0 [36] and the R “Lavaan” package [37]. All tests were two-tailed, with significance level set at 0.05. Categorical variables were reported as frequencies and percentages, while continuous parameters as median and interquartile range.

In our CFA, we used the Robust Weighted Least Squares (RWLS) estimator, which does not assume normally distributed variables and offers the best option for modeling ordinal data in moderately large populations [38]. According to Brown [39], the following four common indices to assess fit of the overall model and to calculate the model adjustment were used: Comparative Fit Index (CFI), Tucker-Lewis Index (TLI), Root Mean Square Error of Approximation (RMSEA), and Standardized Root Mean Square Residual (SRMR). In accordance with the recommendations of Hu and Bentler [40], the following criteria were used: TLI/CFI > 0.90 (accepted fit), RMSEA < 0.08 (accepted fit) and SRMR < 0.08 (good fit). The best fitness of the overall model was represented by higher TLI/CFI values and lower RMSA/SRMR values [40]. Moreover, we calculated the Akaike Information Criterion (AIC), a parsimony correction index to identify the most parsimonious model which fitted the data. Smaller AIC scores indicate the preferred model in terms of comparison to fit and parsimony [41]. We also repeated CFA after 1 year of follow-up (T1) to assess the longitudinal stability of the fit indices for both “Disorganization” factor models investigated.

As no CHR-P participants had a baseline PANSS “Disorganization” dimension score of 0, our statistical analyses were conducted in the CHR-P total sample, as well as in baseline AP medicated and unmedicated subgroups separately. Specifically, Spearman’s rank correlation coefficients and Mann-Whitney U tests were used to examine significant associations of PANSS “disorganization” factor scores with sociodemographic, clinical and psychopathological parameters both at baseline and during the 2-year follow-up period (T2). The Wilcoxon test for repeated measures was also performed to assess the longitudinal stability of PANSS “Disorganization” factor scores across the 2 years of follow-up. Finally, multiple linear regression analyses with PANSS “Disorganization” factor scores as dependent variables and intensity of the specialized PARMS intervention components as independent variables were carried out. In our longitudinal examinations, we used the differences (deltas [Δ]) between T0 and T1 or T2 PANSS scores as primary psychopathological parameters to examine overtime. According to Ver Hoef [42], the delta scores better describe the longitudinal changes and temporal dynamics of psychosis psychopathology compared to T0, T1 and T2 single scores.

## Results

Over the course of the study, 180 CHR-P individuals (90 males, 159 white Caucasians, median age = 20 years old [interquartile range = 16–23 years old]) were recruited. At baseline, 92 (51.1%) of them were taking AP medication. The clinical and sociodemographic characteristics of the total sample and the two subgroups are shown in the Table 1. According to the CAARMS definitions, 140 participants met APS criteria, 30 met BLIPS criteria, and 10 met “Genetic Vulnerability” criteria. In accordance with the DSM-5 diagnostic criteria, the most common primary psychiatric diagnoses were: depressive disorders (*n* = 67), schizotypal personality disorder (*n* = 30), anxiety disorders (*n* = 28), and brief psychotic disorder (*n* = 24) (see Supplementary Materials [Table S1] for details). According to DSM-5 diagnostic criteria [32], psychosis Not Otherwise Specified (NOS) (*n* = 6) was used as a “diagnosis of exclusion” that was applied to clinical manifestations where psychotic symptoms were present without meeting the full criteria for any specific psychotic disorder. This category was also used in presentations in which the clinician did not choose to communicate the specific reason that the manifestation did not meet the criteria for any specific psychotic disorder, including presentations in which there was insufficient or contradictory information to make a more specific diagnosis and cases with psychosis-like symptoms under the threshold of frank psychosis but considered to be of particular high risk for developing a psychotic disorder [43].At baseline, the CHR-P/AP + subgroup showed older age (z = −4.772; *p* = .0001), higher PANSS “Positive Symptoms” factor score (z = −2.099; *p* = .045), and higher prevalence rates of BLIPS (X^2^ = 9.409; *p* = .002) and brief psychotic disorder (X^2^ = 4.311; *p* = .038) in comparison with CHR-P/AP- participants. No inter-group difference in disorganization levels was found.

**Table 1:** Baseline sociodemographic and clinical characteristics in the CHR-P total sample and the two subgroups

Variable	CHR-P total sample (*n* = 180)	CHR-*P*/AP+	CHR-*P*/AP-	X^2^/z	*p*
		Subgroup (*n* = 92)	subgroup (*n* = 88)		
Gender (males)	90 (50.0%)	45 (48.9%)	45 (51.1%)	0.089	0.766
Ethnic group (white Caucasian)	159 (88.3%)	80 (87.0%)	79 (89.8%)	0.346	0.556
Migrant Status	28 (15.6%)	14 (15.2%)	14 (15.9%)	0.016	0.898
Age (at entry)	20 (16–23)	21 (18–23.75)	17 (15–21.75)	−4.722	**0.0001**
Education (in years)	12 (9–13)	12 (9–13)	11 (9.25–13)	−1.659	0.097
DUPS (in weeks)	30 (12–52)	26 (12–49.50)	38 (12–67.50)	0.984	0.325
Past specialist contact	83 (46.1%)	44 (47.8%)	39 (44.3%)	0.223	0.637
Current substance abuse	31 (17.2%)	18 (19.6%)	13 (14.8%)	0.725	0.395
*CHR-P subgroups*					
APS	140 (77.8%)	64 (69.6%)	76 (86.4%)	7.343	**0.007**
BLIPS	30 (16.7%)	23 (12.8%)	7 (8.0%)	9.409	**0.002**
Genetic vulnerability	10 (5.5%)	5 (5.4%)	5 (5.7%)	0.099	0.901
*DSM-5 diagnoses*					
Depressive disorder	67 (37.2%)	32 (34.8%)	35 (39.8%)	0.202	0.857
Schizotypal personality disorder	30 (16.7%)	15 (16.3%)	15 (17.1%)	0.101	0.894
Anxiety disorder	28 (15.5%)	14 (15.2%)	14 (15.9%)	0.088	0.905
Brief psychotic disorder	24 (13.3%)	17 (18.5%)	7 (8.0%)	4.311	**0.038**
Borderline personality disorder	13 (7.2%)	5 (5.4%)	8 (9.1%)	0.874	0.174
Obsessive-compulsive disorder	8 (4.4%)	4 (4.3%)	4 (4.5%)	0.098	0.911
Psychotic disorder NOS	6 (3.3%)	4 (4.2%)	2 (2.3%)	1.074	0.101
Eating disorder	4 (2.4%)	1 (1.1%)	3 (3.4%)	1.221	0.109
AP prescription rate	92 (51.1%)	92 (100.0%0)	0 (0.0%)	–	–
Equivalent dose of CPZ (mg/day)	60 (60–120)	120 (60–225)	0 (0–0)0 (0.0%)	–	–
Risperidone	51 (28.3%)	51 (55.4%)	−0 (0.0%)		
(equivalent dose of CPZ [mg/day])	–	120 (67.5–180)	–		
Olanzapine	20 (11.1%)	20 (21.7%)	0 (0.0%)		
(equivalent dose of CPZ [mg/day])	–	135 (60–300)	–		
Aripiprazole	12 (6.7%)	12 (13.0%)	0 (0.0%)		
(equivalent dose of CPZ [mg/day])	–	120 (97.5–225)	–		
Quetiapine	9 (5.0%)	9 (10.2%)	18 (20.5%)		
(equivalent dose of CPZ [mg/day])	–	120 (105–120)	30 (30–60)		
AD prescription rate	43 (23.9%)	25 (27.2%)	19 (21.6%)	1.117	0.291
Equivalent dose of fluoxetine (mg/day)	30 (20–40)	30 (10–30)	2.5 (1.75–5.75)	−1.561	0.119
BDZ prescription rate	41 (22.8%)	22 (23.9%)		0.498	0.854
Equivalent dose of lorazepam (mg/day)	2 (1–2.5)	1.75 (1–2.5)		−1.309	0.191
			10 (7–12.50)		
*PANSS*			19 (12–24)		
Positive symptoms	10 (8–13)	11.50 (8–13)	14 (11.50–18.50)	−2.099	**0.045**
Negative Symptoms	19 (13–24)	19 (15–24)	15 (11–17.50)	−0.855	0.392
Disorganization	14 (12–19)	14 (12–20)	7 (5–10)	−1.037	0.3
Affect	15 (13–18)	15.50 (13–19)	68 (54.50–80)	−1.301	0.193
Resistance/Excitement-activity	6.50 (5–9)	6 (5–8)		0.659	0.51
Total score	70 (59.50–80)	71 (62–80)	50 (44–55)	−1.265	0.206
GAF score	50 (45.50–54.75)	50 (42–50.75)		−1.946	0.059

*Note* CHR-P = Clinical High Risk for Psychosis; CHR-P/AP + = CHR-P individuals with baseline AP medication; CHR-P/AP- = CHR-P individuals without baseline AP medication; DSM-5 = Diagnostic and Statistical Manual of mental disorders − 5th Edition; DUPS = Duration of Untreated Psychiatric Symptoms; APS = Attenuated Psychotic Symptoms, BLIPS = Brief Limited Intermittent Psychotic Symptoms; GRFD = Genetic Risk Functioning Deterioration syndrome; NOS = Not Otherwise Specified; AP = Antipsychotic; CPZ = Chlorpromazine; AD = Antidepressant; BDZ = Benzodiazepine; PANSS = Positive And Negative Syndrome Scale; GAF = Global Assessment of Functioning; p = statistical significance. Frequencies (and percentages), median (and interquartile range), Chi-squared test (X^2^) and Mann-Whitney test (z) values are reported. Statistically significant p values are in bold. The database did not include more socio-demographic/clinical information for this specific research

Compared to the 5-item factor model identified by Yang and colleagues [28], the 8-item “Disorganization” factor model (18) showed the best fit indices in our population, both at baseline (T0) and after 1 year of follow-up (T1) (see the Table 2 for details on CFA tests and values). Therefore, we used this PANSS factor structure configuration in the remaining statistical analyses of the current research.

**Table 2:** CFA indices of adjustment in the CHR-P total sample (*n* = 180): exploring competitive models of different PANSS “Disorganization” factor configurations

PANSS “disorganization” factor models	χ^2^	CFI	TLI	RMSEA	SRMR	AIC
	(df; *p*)					
*T0*						
8-item factor model	46.194 (20;0.0001)	0.99	0.981	0.126	0.051	1898.166
5-factor model	5.993 (5;0.307)	0.894	0.851	0.473	0.098	2948.676
*T1*						
8-item factor model	67.886 (20;0.0001)	0.945	0.891	0.132	0.078	1837.721
5-factor model	15.638 (5;0.008)	0.914	0.88	0.14	0.109	2687.243

*Note* CFA = Confirmatory Factor Analysis; PANSS = Positive And Negative Syndrome Scale; CHR-P = Clinical High Risk for Psychosis; χ^2^ = Chi-squared test value; df = degrees of freedom; p = statistical significance; T0 = baseline assessment time; T1 = 1-year assessment time; T2 = 2-year assessment time; CFI = Comparative Fit Index; TLI = Tucker-Lewis Index; RMSEA = Root Mean Square Error of Approximation; SRMR = Standardized Root Mean Square Residual; AIC = Akaike Information Criterion; 8-item “Disorganization” factor model including PANSS P2, N5, N7, G5, G10, G11, G13, and G15 items (Shafer & Dazzi, 2019); 5-item factor model including PANSS P2, P5, G9, G11, and G13 items (Yang et al., 2018)

As for *baseline* characteristics of the CHR-P total sample, the PANSS “Disorganization” factor subscore positively correlated with PANSS total score and PANSS “Positive Symptoms” (ρ = 0.284; 0.004), “Negative Symptoms” (ρ = 0.531; *p* = .0001), and “Affect” (ρ = 0.322; *p* = .0001) factor subscores. Moreover, it also negatively correlated with GAF score at entry (ρ = − 0.266; *p* = .003) (Table 3). In the CHR-P/AP + subgroup, PANSS “Disorganization” factor subscore positively correlated with PANSS total score (ρ = 0.841; *p* = .0001) and all the other PANSS factor subscores (see the Table 3 for details). Differently, in CHR-P/AP- participants, it exclusively correlated with PANSS total score (ρ = 0.728; *p* = .0001) and PANSS “Negative Symptoms” factor subscore (ρ = 0.546; *p* = .0001), as well as negatively with GAF score at entry (ρ = − 0.339; *p* = .006).

**Table 3:** Baseline associations between disorganization and sociodemographic/clinical parameters at baseline in the CHR-P total sample and the two subgroups

	PANSS “disorganization” factor score (ρ; *p*)	PANSS “disorganization” factor score (z; *p*)
*Variables in the CHR-P total sample (n = 180)*		
Gender	–	–0.787; 0.431
Ethnic group (white Caucasian)	–	–0.714; 0.475
Migrant status	–	0.312; 0.755
Age (at entry)	0.083; 0.359	–
Education (in years)	–0.005; 0.956	–
DUPS (in weeks)	–0.135; 0.137	–
Past specialist contact	–	–1.028; 0.304
Substance abuse (at entry)	–	1.372; 0.170
APS	–	–0.523; 0.601
BLIPS	–	–0.778; 0.437
PANSS Positive symptoms	0.284; **0.004**	–
PANSS Negative symptoms	0.531; **0.0001**	–
PANS Affect	0.322; **0.0001**	–
PANSS Resistance/Excitement-Activity	0.227; 0.059	–
PANSS total score	0.771; **0.0001**	–
GAF score	–0.266; **0.003**	–
Equivalent dose of chlorpromazine (mg/day)	0.131; 0.149	–
Equivalent dose of fluoxetine (mg/day)	0.017; 0.849	–
Equivalent dose of lorazepam (mg/day)	0.144; 0.111	–
*Variables in the CHR-P/AP+ subgroup (n = 92)*		
Gender	–	−0.380; 0.704
Ethnic group (white Caucasian)	–	−1.177; 0.239
Migrant status	–	1.175; 0.240
Age (at entry)	0.065; 0.628	–
Education (in years)	−0.155; 0.245	–
DUPS (in weeks)	−0.115; 0.389	–
Past specialist contact	–	0.125; 0.901
Substance abuse (at entry)	–	−0.127; 0.903
APS	–	1.282; 0.200
BLIPS	–	−1.168; 0.243
PANSS Positive symptoms	0.340; **0.009**	–
PANSS Negative symptoms	0.502; **0.0001**	–
PANSS Affect	0.378; **0.003**	–
PANSS Resistance/Excitement-Activity	0.393; **0.002**	–
PANSS Total score	0.841; **0.0001**	–
GAF score	−0.156; 0.241	–
Equivalent dose of chlorpromazine (mg/day)	0.194; 0.145	–
Equivalent dose of fluoxetine (mg/day)	−0.141; 0.293	–
Equivalent dose of lorazepam (mg/day)	0.129; 0.336	–
*Variables in the CHR-P/AP- subgroup (n = 88)*		
Gender	–	−0.753; 0.452
Ethnic group (white Caucasian)	–	0.702; 0.711
Migrant status	–	−0.711; 0.477
Age (at entry)	0.092; 0.464	–
Education (in years)	0.115; 0.362	–
DUPS (in weeks)	−0.145; 0.250	–
Past specialist contact	–	−1.586; 0.113
Substance abuse (at entry)	–	1.808; 0.071
APS subgroup	–	−1.868; 0.062
BLIPS subgroup	–	0.381; 0.703
PANSS Positive symptoms	0.247; 0.069	–
PANSS Negative symptoms	0.544; **0.0001**	–
PANSS Affect	0.256; 0.060	–
PANSS Resistance/Excitement-Activity	0.116; 357	–
PANSS total score	0.728; **0.0001**	–
GAF score	−0.339; **0.006**	–
Equivalent dose of fluoxetine (mg/day)	0.146; 247	–
Equivalent dose of lorazepam (mg/day)	0.152; 0.225	–

*Note* CHR-P = Clinical High Risk for Psychosis; AP = Antipsychotic medication; CHR-P/AP + = CHR-P individuals with baseline AP treatment; CHR-P/AP- = CHR-P individuals without baseline AP treatment; PANSS = Positive And Negative Syndrome Scale; DUPS = Duration of Untreated Psychiatric Symptoms; APS = Attenuated Psychotic Symptoms; BLIPS = Brief Limited Intermittent Psychotic Symptoms; GAF = Global Assessment of Functioning; p = statistical significance. Spearman rank correlation (ρ) values (for categorical parameters) and Mann-Whitney U test (z) values (for continuous parameters) are reported. Bonferroni’s corrected p values are reported. Statistically significant p values are in bold

As for attrition rate, 5 (2.8%) CHR-P participants dropped out in the first year of treatment (1 in the CHR-P/AP + subgroup and 4 in the CHR-P/AP- subgroup), actively refusing further contact with the treatment staff and being no longer traceable, while 22 (12.2%) did not complete the second year of intervention (10 in the CHR-P/AP + subgroup and 14 in the CHR-P/AP- subgroup): specifically, 6 of them voluntarily dropped out the PARMS program and 11 prematurely left EIP treatments according to their clinicians (see also Supplementary Materials [Table S2] for further details).

Across the 2 years of our follow-up (Table 4), we found a significant decrease in the PANSS “Disorganization” factor scores in the CHR-P total sample (z = −4.206; *p* = .0001), more relevant between T0 and T1 (z = −3.105; *p* = .002). Notably, this statistically significant longitudinal reduction was confirmed exclusively in the CHR-P/AP + subgroup (T0-T2 z = −4.274, *p* = .0001; T0-T1 z = −3.357, *p* = .001).

**Table 4:** Longitudinal course of PANSS “disorganization” dimension scores in the CHR-P total sample and the two subgroups across the 2-year follow-up period

Variable in the CHR-*P* total sample	T0 (*n* = 180)	T1 (*n* = 175)	T2 (*n* = 153)	T0-T1 (z; *p*)	T0-T2 (z; *p*)	T1-T2 (z; *p*)
PANSS “Disorganization” factor scores	14 (12–19)	13 (10–17)	12 (10–17)	−3.105; **0.002**	−4.206; **0.0001**	−2.816; **0.005**
Variable in the CHR-P/AP + subgroup	T0 (*n* = 92)	T1 (*n* = 91)	T2 (*n* = 83)	T0-T1 (z; p)	T0-T2 (z; p)	T1-T2 (z; p)
PANSS “Disorganization” factor scores	14 (12–20)	13 (10–17)	11.50 (10–16)	−3.357; **0.001**	−4.274; **0.0001**	−2.663; **0.008**
Variable in the CHR-P/AP- subgroup	T0 (*n* = 88)	T1 (*n* = 84)	T2 (*n* = 70)	T0-T1 (z; p)	T0-T2 (z; p)	T1-T2 (z; p)
PANSS “Disorganization” factor scores	14 (11.50–18.50)	14 (10–18)	13 (9.50–18)	− 0.955; 0.340	−1.691; 0.091	−1.479; 139

*Note* PANSS = Positive And Negative Syndrome Scale; CHR-P = Clinical High Risk for Psychosis; AP = Antipsychotic medication; CHR-P/AP + = CHR-P participants with baseline AP treatment; CHR-P/AP- = CHR-P participants without baseline AP treatment; T0 = baseline assessment; T1 = 1-year assessment time; T2 = 2-year assessment time. Median (interquartile range) and Wilcoxon test (z) values are reported. Statistically significant p values are in bold

In the CHR-P total sample, such decrease in disorganization severity levels correlated positively with improvement in GAF score (ρ = − 0.382; *p* = .0001) and in all the other PANSS factor scores, yet negatively with DUPS (ρ = − 0.260; *p* = .004) (see the Table 5 for details on statistic tests and values). This correlation pattern was substantially confirmed in both CHR-P/AP + and CHR-P/AP- subgroups, with the exception of the association with DUPS (see the Table 5 for details). Notably, in all the three CHR-P groups here examined, the most statistically robust correlation coefficients were with improvements in negative symptom levels over time.

**Table 5:** Longitudinal association between disorganization and other clinical parameters in the CHR-P total sample and the two subgroups across the 2-year follow-up period

	T0-T2 PANSS “Disorganization” factor score (ρ) (*p*)	T0-T2 PANSS “Disorganization” factor score (z) (*p*)
*Variables in the CHR-P total sample (n = 153)*		
Gender	–	−1.523; 0.128
Ethnic group (white Caucasian)	–	−0.445; 0.656
Migrant status	–	−1.304; 0.192
Age (at entry)	0.104; 0.252	–
Education (in years)	0.019; 0.838	–
DUPS (in weeks)	−0.260; **0.004**	–
Past specialist contact	–	−0.723; 0.470
Substance abuse (at entry)	–	−0.382; 0.702
APS subgroup	–	−0.818; 0.413
BLIPS subgroup	–	−0.684; 0.494
T0-T2 PANSS “Positive symptoms” factor score	0.389; **0.0001**	–
T0-T2 PANSS “Negative symptoms” factor score	0.608; **0.0001**	–
T0-T2 PANSS “Affect” factor score	0.426; **0.0001**	–
T0-T2 PANSS “Resistance/Excitement-Activity” factor score	0.403; **0.0001**	–
T0-T2 GAF score	−0.382; **0.0001**	–
T0 equivalent dose of chlorpromazine (mg/day)	0.131; 0.149	–
T1 equivalent dose of chlorpromazine (mg/day)	0.279; **0.002**	–
T2 equivalent dose of chlorpromazine (mg/day)	0.238; **0.017**	–
T0 equivalent dose of fluoxetine (mg/day)	0.017; 0.849	–
T1 equivalent dose of fluoxetine (mg/day)	0.001; 0.999	–
T2 equivalent dose of fluoxetine (mg/day)	0.066; 0.513	–
T0 equivalent dose of lorazepam (mg/day)	0.144; 0.111	–
T1 equivalent dose of lorazepam (mag/day)	0.184; 0.065	–
T2 equivalent dose of lorazerpam (mg/day)	0.174; 0.095	–
*Variables in the CHR-P/AP+ subgroup (n = 83)*		
Gender	–	−0.950; 0.342
Ethnic group (white Caucasian)	–	−0.622; 0.534
Migrant status	–	−0.302; 0.763
Age (at entry)	0.092; 0.493	–
Education (in years)	−0.086; 0.521	–
DUPS (in weeks)	−0.255; 0.053	–
Past specialist contact	–	−0.195; 0.845
Substance abuse (at entry)	–	−0.927; 0.354
APS subgroup	–	−1.245; 0.213
BLIPS subgroup	–	−1.084; 0.278
T0-T2 PANSS “Positive symptoms” factor score	0.367; **0.0001**	–
T0-T2 PANSS “Negative symptoms” factor score	0.722; **0.0001**	–
T0-T2 PANSS “Affect” factor score	0.589; **0.0001**	–
T0-T2 PANSS “Resistance/Excitement-Activity” factor score	0.422; **0.001**	–
T0-T2 GAF score	−0.341; **0.015**	–
T0 equivalent dose of chlorpromazine (mg/day)	0.194; 0.145	–
T1 equivalent dose of chlorpromazine (mg/day)	0.246; 0.062	–
T2 equivalent dose of chlorpromazine (mg/day)	0.093; 0.519	–
T0 equivalent dose of fluoxetine (mg/day)	−0.141; 0.293	–
T1 equivalent dose of fluoxetine (mg/day)	–0.171; 0.200	–
T2 equivalent dose of fluoxetine (mg/day)	−0.017; 0.626	–
T0 equivalent dose of lorazepam (mg/day)	0.129; 0.336	–
T1 equivalent dose of lorazepam (mag/day)	0.151; 0.258	–
T2 equivalent dose of lorazerpam (mg/day)	0.120; 0.456	–
*Variables in the CHR-P/AP+ subgroup (n = 70)*		
Gender	–	−1.445; 0.148
Ethnic group (white Caucasian)	–	−0.309; 0.758
Migrant status	–	−1.692; 0.091
Age (at entry)	0.026; 0.838	–
Education (in years)	0.030; 0.813	–
DUPS (in weeks)	−0.235; 0.059	–
Past specialist contact	–	−0.790; 0.430
Substance abuse (at entry)	–	−0.088; 0.930
APS subgroup	–	−0.085; 0.932
BLIPS subgroup	–	−0.819; 0.413
T0-T2 PANSS “Positive symptoms” factor score	−0.380; **0.002**	–
T0-T2 PANSS “Negative symptoms” factor score	0.497; **0.0001**	–
T0-T2 PANSS “Affect” factor score	0.223; 0.074	–
T0-T2 PANSS “Resistance/Excitement-Activity” factor score	0.386; **0.001**	–
T0-T2 GAF score	−0.395; **0.004**	–
T1 equivalent dose of chlorpromazine (mg/day)	0.202; 0.101	–
T2 equivalent dose of chlorpromazine (mg/day)	0.094; 0.527	–
T0 equivalent dose of fluoxetine (mg/day)	0.146; 0.247	–
T1 equivalent dose of fluoxetine (mg/day)	0.143; 0.257	–
T2 equivalent dose of fluoxetine (mg/day)	0.193; 0.175	–
T0 equivalent dose of lorazepam (mg/day)	0.152; 0.225	–
T1 equivalent dose of lorazepam (mag/day)	−0.237; 0.075	–
T2 equivalent dose of lorazerpam (mg/day)	−0.249; 0.075	–

*Note* CHR-P = Clinical High Risk for Psychosis; AP = Antipsychotic medication; CHR-P/AP + = CHR-P participants with baseline AP treatment; CHR-P/AP- = CHR-P participants without baseline AP treatment; PANSS = Positive And Negative Syndrome Scale; DUPS = Duration of Untreated Psychiatric Symptoms; APS = Attenuated Psychotic Symptoms; BLIPS = Brief Limited Intermittent Psychotic Symptoms; T0 = baseline assessment time; T2 = 2-year assessment time; GAF = Global Assessment of Functioning; p = statistical significance. Spearman rank correlation (ρ) values (for categorical parameters) and Mann-Whitney U test (z) values (for continuous parameters) are reported. Bonferroni’s corrected p values are reported. Statistically significant p values are in bold

Finally, our multiple linear regression analysis results in the CHR-P total sample showed that longitudinal improvement in disorganization severity levels during the follow-up was predicted by equivalent doses of AP medication (both at baseline [T0B = 1.444; *p* = .0001] and during the follow-up period [T2B = 1.192; *p* = .023]), and shorter DUPS (B = − 0.027; *p* = .00in3) (Table 6), especially in the first year of treatment. A further negative predictive factor was represented by equivalent doses of benzodiazepine (particularly at entry). This prediction pattern was substantially confirmed in the CHR-P/AP + subgroup (see Supplementary Materials [Table S3] for details), together with evidence of the total number of family psychoeducation sessions showing a statistically significant positive association with the reduction in disorganization levels during the first year of intervention (B coefficient = 0.383; Standard Error = 0.149; *p* = .013).

**Table 6:** PANSS “disorganization” factor scores and their associations with clinical parameters and specialized treatment components of the PARMS program in the CHR-P total sample across the 2-year follow-up period

	B	SE	β	*p*	95% CI		
					Lower	Upper	
*T0-T1 PANSS “Disorganization” factor score (n = 175)*							R^2^ = 0.258 F _[df = 10]_ = 3.901 p = **.0001**
Constant	1.794	0.642	–	0.006	0.522	3.066	
T0 equivalent dose of chlorpromazine (mg/day)	0.836	0.236	0.426	**0.001**	0.369	1.303	
T1 equivalent dose of chlorpromazine (mg/day)	0.55	0.209	0.307	**0.01**	0.136	0.965	
T0 equivalent dose of fluoxetine (mg/day)	−0.005	0.006	−0.091	0.361	−0.017	0.006	
T1 equivalent dose of fluoxetine (mg/day)	0.005	0.005	0.101	0.292	−0.004	0.014	
T0 equivalent dose of lorazepam (mg/day)	−1.098	0.371	−0.439	**0.004**	−1.834	−0.363	
T1 equivalent dose of lorazepam (mag/day)	−1.095	0.473	−0.35	**0.022**	−2.032	−0.158	
T1 number of individual psychotherapy sessions	0.003	0.045	0.008	0.948	−0.086	0.092	
T1 number of family psychoeducation sessions	0.193	0.101	0.226	0.058	−0.007	0.393	
T1 number of case management sessions	−0.014	0.013	−0.09	0.299	−0.04	0.012	
DUPS	−0.021	0.006	−0.279	**0.001**	−0.034	−0.008	
*T0-T2 PANSS “Disorganization” factor score (n = 153)*							R^2^ = 0.294 F _[df = 13]_ = 2.785 p = **.002**
Constant	2.824	1.02	−	0.007	0.797	4.851	
T0 equivalent dose of chlorpromazine (mg/day)	1.444	0.356	0.602	**0.0001**	0.737	2.151	
T1 equivalent dose of chlorpromazine (mg/day)	0.123	0.428	0.057	0.774	−0.727	0.974	
T2 equivalent dose of chlorpromazine (mg/day)	1.192	0.514	−0.426	**0.023**	−2.214	−0.171	
T0 equivalent dose of fluoxetine (mg/day)	−0.008	0.009	−0.115	0.353	−0.025	0.009	
T1 equivalent dose of fluoxetine (mg/day)	0.002	0.008	0.034	0.812	−0.014	0.018	
T2 equivalent dose of fluoxetine (mg/day)	0.011	0.011	0.169	0.302	−0.01	0.033	
T0 equivalent dose of lorazepam (mg/day)	−1.482	0.53	−0.49	**0.006**	−2.536	−0.428	
T1 equivalent dose of lorazepam (mag/day)	1.412	0.844	0.377	0.098	−0.265	3.088	
T2 equivalent dose of lorazerpam (mg/day)	0.159	0.611	0.05	0.795	−1.055	1.373	
T2 number of individual psychotherapy sessions	−0.013	0.03	−0.058	0.658	−0.074	0.047	
T2 number of family psychoeducation sessions	0.007	0.053	0.018	0.89	−0.097	0.112	
T2 number of case management sessionsDUPS	0.001	0.007	0.006	0.947	−0.013	0.014	
	−0.027	0.009	−0.284	**0.003**	−0.044	−0.009	
*T1-T2 PANSS “Disorganization” factor score (n = 153)*							R^2^ = 0.133 F _[df = 13]_ = 1.022 *p* = .438
Constant	0.966	0.614	–	0.119	−0.254	2.185	
T0 equivalent dose of chlorpromazine (mg/day)	0.408	0.214	0.313	0.06	−0.017	0.834	
T1 equivalent dose of chlorpromazine (mg/day)	0.129	0.258	0.11	0.616	−0.383	0.642	
T2 equivalent dose of chlorpromazine (mg/day)	−0.214	0.309	−0.141	0.491	−0.829	0.401	
T0 equivalent dose of fluoxetine (mg/day)	−0.003	0.005	−0.071	0.606	−0.013	0.008	
T1 equivalent dose of fluoxetine (mg/day)	−0.003	0.005	−0.089	0.572	−0.012	0.007	
T2 equivalent dose of fluoxetine (mg/day)	0.011	0.007	0.296	0.105	−0.002	0.024	
T0 equivalent dose of lorazepam (mg/day)	−0.191	0.319	−0.116	0.55	−0.826	0.443	
T1 equivalent dose of lorazepam (mag/day)	0.031	0.508	0.015	0.952	−0.978	1.04	
T2 equivalent dose of lorazerpam (mg/day)	0.183	0.368	0.106	0.62	−0.548	0.914	
T2 number of individual psychotherapy sessions	−0.023	0.018	−0.183	0.207	−0.06	0.013	
T2 number of family psychoeducation sessions	−0.018	0.032	−0.08	0.575	−0.081	0.045	
T2 number of case management sessions	0.003	0.004	0.065	0.54	−0.006	0.011	
DUPS	−0.007	0.005	−0.136	0.196	−0.017	0.004	

*Note* PANSS = Positive And Negative Syndrome Scale; PARMS = Parma At-Risk Mental States; CHR-P = Clinical high Risk for Psychosis. T0 = baseline assessment time; T1 = 1-year assessment time; T2 = 2-year assessment time; DUPS = Duration of Untreated Psychiatric Symptoms; B = regression coefficient, SE = Standard Error, 95% CI = 95% Confident Intervals for B, β = standardized regression coefficient; p = statistical significance, R^2^ = R-squared or coefficient of determination, F = statistic test value for linear regression, df = degrees of freedom. Statistically significant p values are in bold

Although statistically not significant, longitudinal improvement in disorganization among CHR-P/AP- participants was similarly predicted by shorter DUPS and lower equivalent doses of benzodiazepine (see Supplementary Materials [Table S4] for details), as well as by the total number of case management sessions offered during the first year of treatment (B = 0.041; Standard Error = 0.018; *p* = .025). In the CHR-P/AP- subgroup, no predictive role was observed in terms of AP medication prescribed over time. In this respect, only 15 CHR-P/AP- individuals were taken AP drug at the end of our follow-up period.

## Discussion

Although considered as a “core” feature of psychosis, disorganization drew less attention than positive and negative symptoms [13], probably because of early modern models of psychosis psychopathology incorporating disorganized features with “reality distortion” characteristics to establish a positive domain. However, as evidence reported that disorganization in psychosis is an important clinical predictor of poor prognosis and daily functioning deterioration (including in subjects with early psychosis) [19], the aims of this research were to investigate the longitudinal stability of disorganization in a CHR-P population treated within an EIP service along a 2-year follow-up period, and to assess any significant longitudinal association of disorganized dimension with sociodemographic, clinical, and treatment features across the follow-up (also through a comparison between AP medicated and unmedicated CHR-P subgroups). To our knowledge, no perspective research specifically examining disorganization in CHR-P individuals has been reported in the literature to date, especially in terms of treatment response.

First of all, the results of this research suggest that disorganization in CHR-P individuals is closely related to other dimensions of psychosis psychopathology (particularly negative symptoms rather than positive ones) and these connections remain relevant over time. In particular, the privileged and temporally stable link of disorganization with negative features is clearly supported by the evidence of their most statistically robust correlation coefficients observed in all the three CHR-P groups here examined, both at baseline and across the 2 years of follow-up. This seems recall the Bleulerian conceptualization of “loosening of association” as one of the most important (“*fundamental*”) *symptoms* in psychosis (especially in schizophrenia), together with other clinical features currently classified as negative symptoms (i.e., volitional indeterminacy, affective incongruence, and withdrawal from reality) [3]. In the author’s thinking, these psychopathological characteristics were considered as “nuclear” in the psychotic disorder, being stably present (right from the onset of psychotic disorder) [44, 45]. Moreover, as reported in young patients with first episode psychosis [1, 2], the contextual decrease of all these PANSS factor subscores (and PANSS total scores) further supports the validity of disorganization as a *general severity index* both at presentation (i.e., already from the first specialist contact with CHR-P services [also in adolescence]) and over the 2 years of our follow-up. Indeed, in our research, this appeared longitudinally evident both in the CHR-P total sample and in the two subgroups.

The findings of this investigation also suggest a significant, enduring association between disorganization and daily *functioning decline*, already at the enrollment time in specialized CHR-P services (particularly evident in CHR-P/AP- individuals). This further supports the evidence that the most severely disorganized patients with early psychosis have greater difficulties in real-world performance [13, 14]. Moreover, when coupled with the statistically relevant inverse correlation between improvement in disorganized symptoms and DUPS observed in the CHR-P total sample, our results indicate that disorganization could be associated with clinical severity at entry and poorer clinical and functioning remission overtime. These findings suggest that disorganization can be considered as one of the most useful psychopathological indicators to monitor clinical evolution and treatment response in CHR-P individuals (together with negative symptoms and functioning decline). It is therefore necessary to pay special attention to the severity of disorganized symptoms right from the prodromal stage of psychosis, as well as it is urgent to develop specific tools for adequately assessing this relevant dimension in CHR-P samples. Furthermore, our findings confirm the priority of shortening the *DUPS* to improve prognosis and outcomes in the CHR-P population [46, 47].

The results of this investigation showed a longitudinal reduction in disorganization severity levels across the follow-up period in the CHR-P total sample, but exclusively in the CHR-P/AP + subgroup. Indeed, although observed also in CHR-P/AP- participants, this decrease did not reach a statistical significance. As for intervention response in our CHR-P total population, the findings of this research indicate that the longitudinal mitigation of disorganized symptoms is primarily related to shorter DUPS and pharmacological interventions with AP medication (both at entry and across the follow-up). This prediction pattern was substantially confirmed in the CHR-P/AP + subgroup, with the addition of the evidence that the total number of family psychoeducation sessions showed a statistically significant positive association with the reduction in disorganization levels during the first year of intervention. Indeed, it is noteworthy that the relevant effects on this clinical improvement occurred within the first year of treatment (with no relevant additional result observed between T1 and T2). This finding may be related to the fact that the greater intensity of PARMS interventions for CHR-P individuals was provided in the first 12 months. Maintaining at least the same treatment intensity and clinical monitoring during the second year of intervention could thus longitudinally extent its beneficial effects [48, 49].

Despite the recommendations by most international CHR-P guidelines that would substantially avoid the AP use in young individuals at CHR-P [50], our findings suggest a potential usefulness of their prescription for attenuating disorganized features, as well as the importance of starting treatment as soon as indicated and then try to get the most out of the first year of intervention [51]. However, further confirmation studies will be necessary to validate the obtained results, especially on larger CHR-P populations. In particular, these findings should also be interpreted in the light of the evidence that baseline AP exposure in CHR-P population could suggest a condition of immanent risk of transition to psychosis [52–54]. Indeed, as baseline severity illness and DUPS appeared to be actually relevant factors in reduction of disorganization in our CHR-P population, individuals could be already in different disease trajectories to begin. Anyway, the predictive role of shorter DUPS and AP medication in disorganization improvement is substantially confirmed also in the CHR-P/AP + subgroups. Moreover, in all our three CHR-P groups, another predictive factor in disorganization reduction is represented by lower equivalent doses of benzodiazepine (both at baseline and across the first year of treatment). Although not immediately intuitive, these findings could be due to a common prescription pattern that favors AP medications over benzodiazepines in CHR-P individuals with predominant disorganized symptoms or more severe general psychopathology. In this respect, compared to CHR-P/AP- at baseline, CHR-P/AP + participants showed higher prevalence of BLIPS, brief psychotic disorder, and emergency room as source of referral.

Finally, an interesting role in predicting disorganization reduction (at least during the first year of treatment) seems also to be played by some psychosocial interventions offered within the PARMS program (specifically by family psychoeducation in the CHR-P/AP + subgroup and case management in the CHR-/AP- subgroup). Overall considered, these results confirm the potential usefulness of combining pharmacological and psychosocial treatments in young individuals with early psychosis.

## Limitations

Strength of this investigation was that we examined CHR-P subjects within a real-world setting, primarily aimed at delivering optimal clinical care pathways within community mental healthcare services. However, our results are mainly generalizable to similar samples. Another limitation that should also be acknowledged was that this investigation was conducted within a CHR-P program that did not specifically focus on disorganization. Psychometric assessment of major psychopathology was performed with the PANSS and the CAARMS, which are clinical interviews frequently used in individuals with early psychosis, but not originally developed for evaluating the disorganization dimension of psychosis. Lacking instruments to specifically assess disorganization in the early stage of psychosis and consequently having to use psychopathological domains derived from factor analysis methods, future research aimed at developing and validating specific psychometric scales for disorganized features are absolutely desirable and necessary. However, given the widespread use of the PANSS in CHR-P populations [20], our findings have the potential to be replicated in other CHR-P samples and offer a lead to further evaluate disorganization and its treatment response in the early phase of psychosis. This is of primary importance, since research on this topic is still scarce and disorganized symptoms have detrimental effects both on daily functioning and real-world performance.

Another limitation is that we could not evaluate the potential link between disorganization and neurocognitive functioning (although recent evidence seems to suggest that they are separate dimensions in patients with first episode psychosis) [13]. Thus, further investigations examining this potential association in CHR-P subjects are needed. However, in the current study, known intellectual disability was among exclusion criteria.

A final limitation of this study is related to the restrictions on AP medication. Indeed, we exclusively used a time restriction on AP medication (i.e., less than 4 weeks) to select our CHR-P participants and to classify them into the two pharmacological subgroups. No dosage restriction was used, but having received 20 mg/day of olanzapine for 2 weeks is probably something different than having received 25 mg/day of quetiapine for 5 weeks. Therefore, a detailed prospective analysis on AP prescription and medication in larger CHR-P populations is needed.

## Conclusions

Disorganization is a clinically relevant dimension in CHR-P individuals (as well as it is in young patients with first episode psychosis) [2]. In particular, it represents a longitudinally stable psychopathological index of general clinical severity (right from the recruitment in specialized CHR-P programs) and maintains significant enduring relationships especially with daily functioning decline and negative symptoms. However, longitudinal improvement in disorganization severity levels seems to be related to shorter DUPS and AP prescription at baseline, especially during the first year of treatment. More clinical attention and targeted interventions on disorganized symptoms in young individuals at CHR-P are thus recommended, especially in order to reach better clinical outcomes and prognosis. Future studies based on longer-term observation are needed to progress and replicate our findings, as well as to investigate any misuse/overuse of antipsychotic medications [55]. In particular, the time has come when the problem of disorganization should be observed by paying attention to its potential inflammatory effect [56].

## Electronic supplementary material

Below is the link to the electronic supplementary material.

Supplementary Material 1
