# Supplementary material for: Disorganization in individuals at clinical high risk for psychosis: psychopathology and treatment response
Source: Eur Arch Psychiatry Clin Neurosci. 2024 Jun 25;275(3):921–35. doi: 10.1007/s00406-024-01855-3 (PMC11947017; doi:10.1007/s00406-024-01855-3)
Supplement: Supplementary file 1 — Supplementary Material 1 [file 406_2024_1855_MOESM1_ESM.docx]

Table S1 – Treatment components of the PARMS program.

| Within the PARMS protocol, CHR-P individuals were assigned to a multi-disciplinary team (consisting of a clinical psychologist, an early rehabilitation case manager, and a psychiatrist) within 3-4 weeks.  In accordance with current official guidelines on “Early Intervention in Psychosis” (EIP) (Schmidt et al., 2015; RER, 2023), Antipsychotic (AP) drug prescription should be proposed when individuals at CHR-P (a) showed a sudden decline in daily functioning, (b) had a rapid escalation to overt psychotic symptoms, (c) showed an immediate risk of suicide or severe violence, or (d) did not adequately respond to any other psychosocial interventions. As a first-line pharmacological treatment, low-dose second-generation AP medication was indicated (Raballo et al., 2021). Antidepressant and benzodiazepine medication could be prescribed for anxiety, depressive symptoms, and/or insomnia.  Individual psychotherapy was shaped on the model suggested by Van der Gaag and colleagues (2012) for CHR-P individuals. At least 15 sessions per CHR-P subject (each lasting 60 minutes) were offered during the first year of treatment (Azzali et al., 2022). Booster sessions were also provided in the second year according to specific clinical needs.  Family psychoeducation was adapted on the model developed by McFarlane and co-workers (2012) for CHR-P individuals. In the first year of treatment, at least 10 psychoeducational sessions were offered to each family (Pelizza et al., 2019). Booster sessions could also be provided in the second year according to specific family problems or clinical needs.  Finally, each individual/family had a dedicated case-manager to coordinate all interventions that have been planned, especially those promoting a recovery-oriented early rehabilitation (such as “patient-tailored” care pathways specifically designed for encouraging functional recovery, social inclusion and job, also using models inspired by supported employment and community care) (Pelizza et al., 2020; Ficarelli et al., 2021). At least 24 sessions per CHR-P subject (each lasting 60 minutes) were provided along the 2 years of follow-up. |
| --- |

Note. PARMS = Parma At-Risk Mental States; CHR-P = Clinical High Risk for Psychosis.

References

+) Schmidt SJ, Schultze-Lutter F, Schimmelmann BG, Maric NP, Salokangas RK, Riecher-Rössler A, van der Gaag M, Meneghelli A, Nordentoft M, Marshall M, Morrison A, Raballo A, Klosterkötter J, Ruhrmann S (2015) EPA guidance on the early intervention in clinical high-risk states of psychoses. Eur Psychiatry 30: 388-404. <https://doi.org/10.1016/j.eurpsy.2015.01.013>.

+) Regione Emilia-Romagna (RER) (2023) Raccomandazioni regionali per la promozione della salute e del benessere in persone all’esordio psicotico, II edition. Centro Stampa della Regione Emilia-Romagna, Bologna.

+) Raballo A, Poletti M, Preti A (2021) Antipsychotic treatment in clinical high risk for psychosis: protective, iatrogenic or further risk flag? Aust N Z J Psychiatry 55: 442-444. https://doi.org/10.1177/0004867420984836.

+) Van der Gaag M, Nieman DH, Rietdijk J, Dragt S, Ising HK, Klaassen RM, Koeter M, Cuijpers P, Wunderink L, Linszen DH (2012) Cognitive behavioral therapy for subjects at ultrahigh risk for developing psychosis: a randomized controlled clinical trial. Schizophr Bull 38: 1180-1188. <https://doi.org/10.1093/schbul/sbs105>.

+) Azzali S, Pelizza L, Scazza I, Paterlini F, Garlassi S, Chiri LR, Poletti M, Pupo S, Raballo A (2022) Examining subjective experience of aberrant salience in young individuals at ultra-high risk (UHR) of psychosis: a 1-year longitudinal study. Schizophr Res 241: 52-58. <https://doi.org/10.1016/j.schres.2021.12.025>.

+) McFarlane WR, Lynch S, Melton R (2012) Family psychoeducation in clinical high risk and first-episode psychosis. Adolesc Psychiatry 2: 182-194. <https://doi.org/10.2174/2210676611202020182>.

+) Pelizza L, Azzali S, Garlassi S, Scazza I, Paterlini F, Chiri LR, Poletti M, Pupo S, Raballo A (2019) Examining subjective experience of social cognition in early psychosis: validation of the Italian version of the GEOPTE scale in an adolescent and young adult clinical sample. J Psychopathol 25: 220-230. <https://old.jpsychopathol.it/wp-content/uploads/2019/12/06_Pelizza-1.pdf>.

+) Pelizza L, Leuci E, Landi G, Quattrone E, Azzali S, Pelosi A, Ceroni P, Soncini C, Daolio MC, Dall’Aglio R, Paulillo G, Pellegrini C, Raballo A, Pellegrini P (2020) The “Personal Health Budget” intervention model in early psychosis: preliminary findings from the Parma experience. J Psychopathol 26: 209-217. <https://doi.org/10.36148/2284-0249-359>.

+) Ficarelli ML Troisi E, Vignali E, Artoni S, Franzini MC, Montanaro S, Andreoli MV, Marangoni S, Ciampà E, Erlicher D, Pupo S, Pelizza L (2021) Implementing individual and placement support for patients with severe mental illness: findings from the real world. J Psychopathol 27: 71-80. <https://doi:org/10.36148/2284-0249-346>.

Table S1 – Baseline sociodemographic and clinical characteristics in the CHR-P total sample (n = 180) and the most frequent DSM-5 diagnostic subgroups.

| Variable | CHR-P total sample  (n=180) | Depressive disorders  (n=67) | Schizotypal personality disorders  (n = 30) | Anxiety disorders  (n=28) | Brief psychotic disorder  (n=24) | Borderline personality disorders (n=13) | Other DSM-5 diagnoses  (n=18) |
| --- | --- | --- | --- | --- | --- | --- | --- |
| Gender (males)  Ethnic group (white Caucasian)  Migrant Status  Age (at entry)  Education (in years)  DUPS (in weeks)  Past specialist contact  Current substance abuse  *CHR-P subgroups*  APS BLIPS  Genetic vulnerability  *DSM-5 diagnoses*  Depressive disorder  Schizotypal personality disorder  Anxiety disorder  Brief psychotic disorder  Borderline personality disorder  Obsessive-compulsive disorder  Psychotic disorder NOS  Eating disorder  AP prescription rate  Risperidone  Olanzapine  Aripiprazole  Quetiapine  AD prescription rate  BDZ prescription rate | 90 (50.0%)  159 (88.3%)  28 (15.6%)  20 (16-23)  12 (9-13)  30 (12-52)  83 (46.1%)  31 (17.2%)  140 (77.8%)  30 (16.7%)  10 (5.5%)  67 (37.2%)  30 (16.7%)  28 (15.5%)  24 (13.3%)  13 (7.2%)  8 (4.4%)  6 (3.3%)  4 (2.4%)  92 (51.1%)  51 (28.3%)  20 (11.1%)  12 (6.7%)  9 (5.0%)  43 (23.9%)  41 (22.8%) | 27 (40.3%)  56 (83.6%)  12 (17.9%)  18 (15-18)  11 (9.75-13)  31 (12-52)  30 (44.8%)  12 (17.9%)  64 (95.5%)  0 (0.0%)  3 (4.5%)  67 (100.0%)  -  -  -  -  -  -  -  -  29 (43.2%)  -  -  -  -  18 (26.8%)  15 (22.4%) | 11 (36.7%)  29 (96.7%)  1 (3.3%)  19.5 (17-23.5)  12 (10-13)  24 (12-48)  15 (50.0%)  5 (16.7%)  23 (76.7%)  1 (3.3%)  6 (20.0%)  -  -  30 (100.0%)  -  -  -  -  -  -  17 (56.7%)  -  -  -  -  3 (10.0%)  1 (3.3%) | 16 (57.1%)  26 (92.8%)  2 (7.1%)  18 (14-25)  10 (8-13)  24 (8-49)  14 (50.0%)  5 (17.8%)  26 (92.5%)  0 (0.0%)  2 (7.1%)  -  -  -  28 (100.0%)  -  -  -  -  -  14 (50.0%)  -  -  -  -  4 (14.3%)  4 (14.3) | 16 (66.7%)  21 (87.5%)  6 (25.0%)  22 (19-23)  12 (8-13)  24.5 (17-46)  10 (41.7%)  5 (20.8%)  4 (16.7%)  20 (83.3%)  0 (0.0%)  -  -  -  -  24 (100.0%)  -  -  -  -  17 (70.8%)  -  -  -  -  4 (16.7%)  7 (29.2%) | 10 (76.9%)  12 (92.3%)  2 (15.4%)  21 (20-23.5)  13 (10-13)  48 (14.5-156)  8 (61.5%)  3 (23.1%)  12 (92.3%)  0 (0.0%)  1 (7.7%)  -  -  -  -  -  13 (100.0%)  -  -  -  3 (23.1%)  -  -  -  -  3 (23.1%)  2 (15.4%) | 10 (55.5%)  15 (83.3%)  5 (27.8%)  20 (16-23)  12 (10-10)  30 (12-48)  6 (33.3%)  1 (5.5%)  9 (50.0%)  9 (50.0%)  0 (0.0%)  -  -  -  -  -  -  8 (44.4%)  6 (33.3%)  4 (22.3%)  12 (66.6%)  -  -  -  -  11 (61.1%)  12 (66.6%) |

Note. CHR-P = Clinical High Risk for Psychosis; DSM-5 = Diagnostic and Statistical Manual of mental disorders - 5^th^ Edition; DUPS = Duration of Untreated Psychiatric Symptoms; APS = Attenuated Psychotic Symptoms, BLIPS = Brief Limited Intermittent Psychotic Symptoms; GRFD = Genetic Risk Functioning Deterioration syndrome; NOS = Not Otherwise Specified; AP = Antipsychotic; AD = Antidepressant; BDZ = Benzodiazepine. Frequencies (and percentages) and median (interquartile range) are reported. The database did not include more socio-demographic/clinical information for this specific research.

Table S2 – Sociodemographic and clinical characteristics of CHR-P participants who dropped out the PARMS program during the 2-years of follow-up.

| Variable | CHR-P participants who dropped out the PARMS program  (n = 27) |
| --- | --- |
| Gender (males)  Ethnic group (white Caucasian)  Migrant Status  Age (at entry)  Education (in years)  DUPS (in weeks)  Past specialist contact  Current substance abuse  *CHR-P subgroups*  APS BLIPS  Genetic vulnerability  *DSM-5 diagnoses*  Depressive disorder  Anxiety disorder  Schizotypal personality disorder  Brief psychotic disorder  Borderline personality disorder  Psychotic disorder NOS  AP prescription rate  AD prescription rate  BDZ prescription rate  *PANSS*  Positive symptoms  Negative Symptoms  Disorganization  Affect  Resistance/Excitement-activity  Total score  GAF score | 13 (48.1%)  22 (81.5%)  8 (29.6%)  18 (16-23)  11 (9-12)  24 (10-56)  13 (48.1%)  4 (14.8%)  16 (59.3%)  8 (29.6%)  3 (11.1%)  13 (48.1%)  10 (37.1)  1 (3.7%)  1 (3.7%)  1 (3.7%)  1 (3.7%)  9 (33.3%)  1 (3.7%)  4 (14.8%)  9.50 (7.75-13.25)  17.50 (12-22.25)  12 (11-16.25)  12 (9.50-17.50)  8 (4.75-11.25)  65.50 (51-78.50)  50 (48-57) |

Note. CHR-P = Clinical High Risk for Psychosis; PARMS = Parma At-Risk Mental States; DSM-5 = Diagnostic and Statistical Manual of mental disorders - 5^th^ Edition; DUPS = Duration of Untreated Psychiatric Symptoms; APS = Attenuated Psychotic Symptoms, BLIPS = Brief Limited Intermittent Psychotic Symptoms; GRFD = Genetic Risk Functioning Deterioration syndrome; NOS = Not Otherwise Specified; AP = Antipsychotic; AD = Antidepressant; BDZ = Benzodiazepine; PANSS = Positive And Negative Syndrome Scale; GAF = Global Assessment of Functioning; p = statistical significance. Frequencies (and percentages), median (and interquartile range) are reported. The database did not include more socio-demographic/clinical information for this specific research.

Table S3 - PANSS “Disorganization” factor scores and their associations with clinical parameters and specialized treatment components of the PARMS program in the CHR-P/AP+ subgroup across the 2-year follow-up period.

| T0-T1 PANSS “Disorganization” factor score  (n = 92) | B | SE | β | p | 95% CI  Lower Upper | | R^2^ = .367  F _[df = 10]_ = 2.730  p = **.010** |
| --- | --- | --- | --- | --- | --- | --- | --- |
| Constant  T0 equivalent dose of chlorpromazine (mg/day)  T1 equivalent dose of chlorpromazine (mg/day)  T0 equivalent dose of fluoxetine (mg/day)  T1 equivalent dose of fluoxetine (mg/day)  T0 equivalent dose of lorazepam (mg/day)  T1 equivalent dose of lorazepam (mag/day)  T1 number of individual psychotherapy sessions  T1 number of family psychoeducation sessions  T1 number of case management sessions  DUPS | 2.396  .308  .474  -.007  .012  -.996  1.266  -.016  .383  .011  -.025 | 1.331  .323  .244  .009  .006  .482  .640  .061  .149  .019  .012 | -  .143  .286  -.097  .233  -.422  .416  -.041  .421  .070  -.267 | ,048  .344  **.048**  .460  .066  **.044**  .054  .800  **.013**  .576  **.040** | -.282  -.341  .018  -.025  -.001  -1.966  -.021  -.139  .083  -.028  -.048 | 5.075  .958  .965  .011  .025  -.026  2.553  .108  .682  .050  -.001 |  |

| T0-T2 PANSS “Disorganization” factor score  (n = 91) | B | SE | β | p | 95% CI  Lower Upper | | R^2^ = .434  F _[df = 13]_ = 2.124  p = **.037** |
| --- | --- | --- | --- | --- | --- | --- | --- |
| Constant  T0 equivalent dose of chlorpromazine (mg/day)  T1 equivalent dose of chlorpromazine (mg/day)  T2 equivalent dose of chlorpromazine (mg/day)  T0 equivalent dose of fluoxetine (mg/day)  T1 equivalent dose of fluoxetine (mg/day)  T2 equivalent dose of fluoxetine (mg/day)  T0 equivalent dose of lorazepam (mg/day)  T1 equivalent dose of lorazepam (mag/day)  T2 equivalent dose of lorazerpam (mg/day)  T2 number of individual psychotherapy sessions  T2 number of family psychoeducation sessions  T2 number of case management sessions  DUPS | 4.933  .976  .209  1.320  -.031  -.002  .037  -1.451  2.593  -.744  -.031  .038  .014  -.033 | 1.866  .423  .450  .587  .013  .008  .016  .591  1.065  .729  .039  .065  .010  .014 | -  .421  .116  .561  -.416  -.044  .429  -.580  .811  -.294  -.145  .108  .205  -.335 | .012  **.027**  .645  **.031**  .063  .767  .065  **.019**  .060  .314  .431  .559  .162  **.022** | 1.150  .119  -.705  .128  -.057  -.019  .005  -2.649  .432  -2.221  -.110  -.094  -.006  -.061 | 8.717  1.833  1.123  2.511  -.005  .014  .068  -.253  4.754  .734  .048  .171  .034  -.005 |  |

| T1-T2 PANSS “Disorganization” factor score  (n = 83) | B | SE | β | p | 95% CI  Lower Upper | | R^2^ = .262  F _[df = 13]_ = .982  p = .487 |
| --- | --- | --- | --- | --- | --- | --- | --- |
| Constant  T0 equivalent dose of chlorpromazine (mg/day)  T1 equivalent dose of chlorpromazine (mg/day)  T2 equivalent dose of chlorpromazine (mg/day)  T0 equivalent dose of fluoxetine (mg/day)  T1 equivalent dose of fluoxetine (mg/day)  T2 equivalent dose of fluoxetine (mg/day)  T0 equivalent dose of lorazepam (mg/day)  T1 equivalent dose of lorazepam (mag/day)  T2 equivalent dose of lorazepam (mg/day)  T2 number of individual psychotherapy sessions  T2 number of family psychoeducation sessions  T2 number of case management sessions  DUPS | 1.681  .421  .132  -.147  -.007  -.007  .005  -.146  .357  -.166  -.028  -.040  .004  -.009 | 1.166  .264  .282  .367  .008  .005  .010  .369  .667  .455  .024  .041  .006  .009 | -  .332  .134  -.114  -.165  -.238  .116  -.106  .204  -.120  -.237  -.207  .117  -.169 | .158  .119  .643  .691  .415  .165  .585  .695  .596  .718  .263  .330  .482  .300 | -.685  -.114  -.439  -.892  -.023  -.017  -.014  -.895  -.994  -1.089  -.077  -.123  -.008  -.027 | 4.046  .957  .703  .598  .010  .003  .025  .603  1.707  .758  .022  .042  .017  .008 |  |

Note – PANSS = Positive And Negative Syndrome Scale; PARMS = Parma At-Risk Mental States; CHR-P = Clinical high Risk for Psychosis; AP = Antipsychotic medication; CHR-P/AP+ = CHR-P participants with baseline AP treatment; T0 = baseline assessment time; T1 = 1-year assessment time; T2 = 2-year assessment time; DUPS = Duration of Untreated Psychiatric Symptoms; B = regression coefficient, SE = Standard Error, 95% CI = 95% Confident Intervals for B, β = standardized regression coefficient; p = statistical significance, R^2^ = R-squared or coefficient of determination, F = statistic test value for linear regression, df = degrees of freedom. Statistically significant p values are in bold.

Table S4 - PANSS “Disorganization” factor scores and their associations with clinical parameters and specialized treatment components of the PARMS program in the CHR-P/AP- subgroup across the 2-year follow-up period.

| T0-T1 PANSS “Disorganization” factor score  (n = 88) | B | SE | β | p | 95% CI  Lower Upper | | R^2^ = .254  F _[df = 9]_ = 2.077  p = **.048** |
| --- | --- | --- | --- | --- | --- | --- | --- |
| Constant  T1 equivalent dose of chlorpromazine (mg/day)  T0 equivalent dose of fluoxetine (mg/day)  T1 equivalent dose of fluoxetine (mg/day)  T0 equivalent dose of lorazepam (mg/day)  T1 equivalent dose of lorazepam (mag/day)  T1 number of individual psychotherapy sessions  T1 number of family psychoeducation sessions  T1 number of case management sessions  DUPS | 2.308  -1.555  .001  -.003  -2.083  -2.254  -.029  .163  .041  -.020 | .725  .628  .009  .007  .833  .945  .076  .152  .018  .007 | -  -.364  .006  -.081  -.738  -.715  -.074  .215  .290  -.330 | .002  .096  .977  .655  **.015**  **.020**  .707  .288  **.025**  **.010** | .855  -2.813  -.017  -.017  -3.753  -4.147  -.180  -.141  .005  -.034 | 3.761  -.297  .018  .011  -.412  -.361  .123  .467  .076  -.005 |  |

| T0-T2 PANSS “Disorganization” factor score  (n = 84) | B | SE | β | p | 95% CI  Lower Upper | | R^2^ = .392  F _[df = 12]_ = 2.038  p = **.048** |
| --- | --- | --- | --- | --- | --- | --- | --- |
| Constant  T1 equivalent dose of chlorpromazine (mg/day)  T2 equivalent dose of chlorpromazine (mg/day)  T0 equivalent dose of fluoxetine (mg/day)  T1 equivalent dose of fluoxetine (mg/day)  T2 equivalent dose of fluoxetine (mg/day)  T0 equivalent dose of lorazepam (mg/day)  T1 equivalent dose of lorazepam (mag/day)  T2 equivalent dose of lorazepam (mg/day)  T2 number of individual psychotherapy sessions  T2 number of family psychoeducation sessions  T2 number of case management sessions  DUPS | 2.419  1.516  -3.931  .018  .042  -.045  -4.674  1.482  -3.978  .005  .011  -.010  -.030 | 1.316  2.939  2.644  .014  .028  .029  1.660  2.523  1.752  .053  .091  .009  .011 | -  .264  -.787  .281  .787  -.827  -1.182  .342  -.900  .018  .024  -.147  -.357 | .044  .609  .145  .211  .146  .127  **.008**  .561  .059  .926  .904  .276  **.012** | -.246  -4.434  -9.283  -.010  -.015  -.104  -8.033  -3.626  -7.526  -.102  -.173  -.029  -.053 | 5.084  7.466  1.422  .046  .100  .013  -1.314  6.590  -.431  .112  .195  .008  -.007 |  |

| T1-T2 PANSS “Disorganization” factor score  (n = 70) | B | SE | β | p | 95% CI  Lower Upper | | R^2^ = .251  F _[df = 12]_ = .872  p = .421 |
| --- | --- | --- | --- | --- | --- | --- | --- |
| Constant  T1 equivalent dose of chlorpromazine (mg/day)  T2 equivalent dose of chlorpromazine (mg/day)  T0 equivalent dose of fluoxetine (mg/day)  T1 equivalent dose of fluoxetine (mg/day)  T2 equivalent dose of fluoxetine (mg/day)  T0 equivalent dose of lorazepam (mg/day)  T1 equivalent dose of lorazepam (mag/day)  T2 equivalent dose of lorazepam (mg/day)  T2 number of individual psychotherapy sessions  T2 number of family psychoeducation sessions  T2 number of case management sessions  DUPS | .925  3.584  -4.335  .009  .012  -.005  -1.201  -2.451  3.990  -.012  -.004  -.001  -.011 | .724  1.617  1.454  .008  .016  .016  .913  1.388  .964  .029  .050  .005  .006 | -  1.076  -1.496  .244  .389  -.159  -.523  -.976  1.555  -.073  -016  -.025  -.216 | .209  .063  .055  .252  .444  .754  .196  .085  .061  .691  .933  .845  .100 | -.541  .312  -7.279  -.007  -.020  -.037  -3.049  -5.261  2.039  -.070  -.105  -.011  -.023 | 2.391  6.857  -1.391  .024  .044  .027  .647  .358  5.941  .047  .097  .009  .002 |  |

Note – PANSS = Positive And Negative Syndrome Scale; PARMS = Parma At-Risk Mental States; CHR-P = Clinical high Risk for Psychosis; AP = Antipsychotic medication; CHR-P/AP- = CHR-P participants without AP treatment; T0 = baseline assessment time; T1 = 1-year assessment time; T2 = 2-year assessment time; DUPS = Duration of Untreated Psychiatric Symptoms; B = regression coefficient, SE = Standard Error, 95% CI = 95% Confident Intervals for B, β = standardized regression coefficient; p = statistical significance, R^2^ = R-squared or coefficient of determination, F = statistic test value for linear regression, df = degrees of freedom. Statistically significant p values are in bold.
